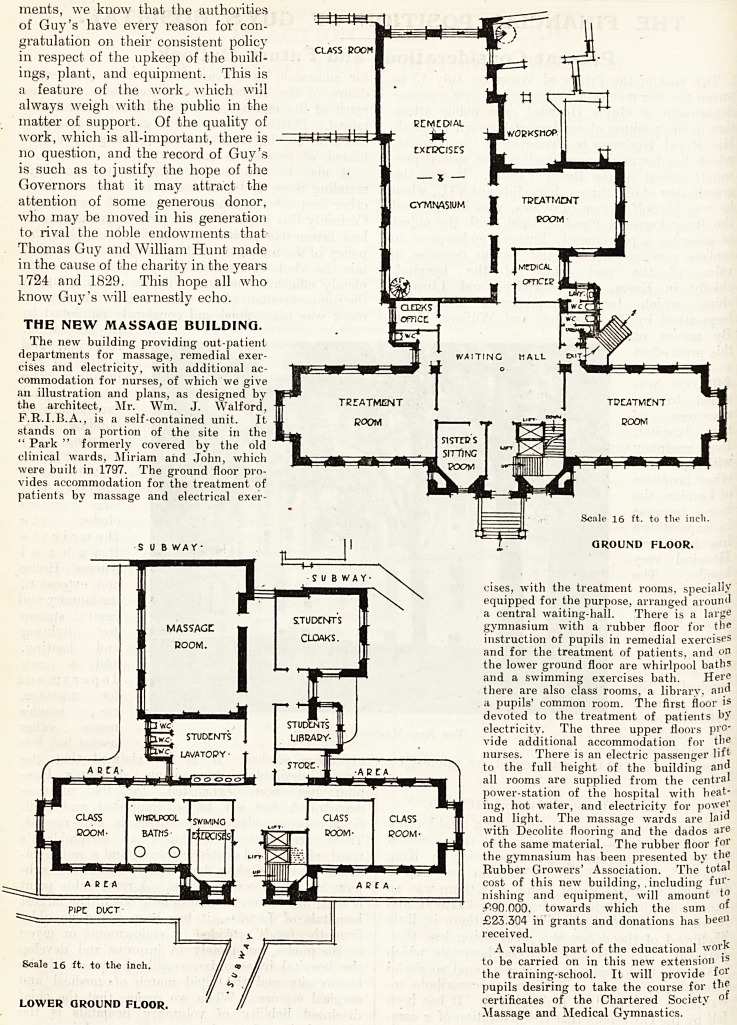# Present Considerations and Future Outlook

**Published:** 1921-07-16

**Authors:** 


					Joly 16, 1921. THE HOSPITAL. 261
THE FINANCIAL POSITION OF GUY'S HOSPITAL.
Present Considerations and Future Outlook.
The visit of the Prince of Wales on July 15 to
unveil the war memorial and open the new massage
department at Guy's Hospital calls public atten-
tion to the position of our largest borough hospital.
His Royal Highness is President of the hospital,
Which has for many years enjoyed the special per-
sonal interest of the Royal Family. It was the
grandfather of our Prince, King Edward VII., when
he was himself Prince of Wales, who inaugurated
the Re-endowment Fund in 1896, with the object
of
securing a permanently invested endowment to
replace revenue lost by the serious decrease in
Value of the real estate of the hospital,
chiefly in Essex, Herefordshire, and Lincoln-
shire, which largely represented the funds
bequeathed by Thomas Guy and William Hunt.
Sy means of
this great effort
a sum of
^400,000 was
added to the
resources of
the hospital.
In company
With all the
other hospitals
?f London, the
huge increase
'n expenditure
has hit Guy's
Hospital very
hardly. The
annual outlay
has more than
doubled
between 1914
and 19 20,
when it
Cached the
large sum of
?163,000, this
not including
e x t inordinary
expenditure of ?2,081 represented by interest on
loans.
An Annual Expenditure of ?163,000.
To meet the expenses of last year a total income
?f ?144,000 was forthcoming, but this included
?7,000 from the emergency distribution by King
-Edward's Hospital Fund for London and ?2,200
from the National Relief Fund. As there was an
^cess of expenditure over income of ?21,000, and
in view of the non-recurrent gifts, there is little
^kelihood of the deficit this year being less than
?30,000. Guy's is one of those hospitals which
has not yet adopted the expedient found so useful
elsewhere of requiring patients to contribute to-
wards the cost of their maintenance. It has been
felt by the Governors that the institution of a com-
pulsory monetary charge to patients as a condition
for admission is at variance with the expressed
desire of the founder and inconsistent with the
spirit of the charity as it has hitherto been main-
tained. Patients are invited to give, and in many
cases do so, but the sum forthcoming from this,
branch of revenue is small.
It may be said that Guy's Hospital, notwith-
standing these facts, is not worse off than many
other hospitals, and possibly better off than some.
Certainly this hospital would have been in a much
less favourable position had it not been that the
policy of the management has always been to main-
tain the whole of the buildings and plant in a com-
pletely efficient condition. To ensure this, up to
the commencement of the war, a large works depart-
ment was maintained and constantly employed in
re - decorating,
repairing, and
adding to the
building. The
total capital
expenditure on
building, re-
novating, etc.,
from 1895 to
19 2 0 was
?366,000. This
large sum in-
cludes the
Henriette
R a p h a el
Nurses' Home
and extension,
the laundry and
central station
for lighting
and heating,
and a new
department
for massage,
etc., besides
many other
useful but less
expensive works. It is not claimed that the
list of desirable improvements at Guy's Hos-
pital has been exhausted, for the massage-
department has yet to he completed and addi-
tional accommodation for nurses is still needed.
There are also such things as a children's
ward, clinical and obstetric wards, and a re-organi-
sation of the kitchen department, which are sub-
jects for future consideration. A remarkable point
is that, in addition to maintaining one of the largest
hospitals of London, it has been found possible,
from the funds provided by endowments or given
by the public, completely to improve and develop
the hospital in accordance with the needs of the
community and the rapid march of medical and
surgical science. When we realise that the chief
disclosed liability of voluntary hospitals is the
accumulation of overdue reparations and improve-
KW*M'
The New Massage Building.
?262 THE HOSPITAL. July 16, 1921.
ments, we know that the authorities
of Guy's have every reason for con-
gratulation on their consistent policy
in respect of the upkeep of the build-
ings, plant, and equipment. This is
a feature of the work., which will
always weigh with the public in the
matter of support. Of the quality of
work, which is all-important, there is
no question, and the record of Guy's
is such as to justify the hope of the
Governors that it may attract the
attention of some generous donor,
who may be moved in his generation
to rival the noble endowments that
Thomas Guy and William Hunt made
in the cause of the charity in the years
1724 and 1829. This hope all who
know Guy's will earnestly echo.
THE NEW MASSAGE BUILDING.
The new building providing out-patient
departments for massage, remedial exer-
cises and electricity, with additional ac-
commodation for nurses, of which we give
an illustration and plans, as designed by
the architect, Mr. Wm. J. Walford,
F.R.I.B.A., is a self-contained unit. It
stands on a portion of the site in the
" Park " formerly covered by the old
clinical wards, Miriam and John, which
were built in 1797. The ground floor pro-
vides accommodation for the treatment of
patients by massage and electrical exer-
cises, with the treatment rooms, specially
equipped for the purpose, arranged around
a central waiting-hall. There is a large
gymnasium with a rubber floor for the
instruction of pupils in remedial exercises
and for the treatment of patients, and on
the lower ground floor are whirlpool baths
and a swimming exercises bath. Here
there are also class rooms, a library, and
a pupils' common room. The first floor is
devoted to the treatment of patients by
electricity. The three upper floors pro-
vide additional accommodation for the
nurses. There is an electric passenger lift-
to the full height of the building and
all rooms are supplied from the central
power-station of the hospital with heat-
ing, hot water, and electricity for power
and light. The massage wards are laid
with Decolite flooring and the dados ai'e
of the same material. The rubber floor foi'
the gymnasium has been presented by the
Rubber Growers' Association. The total
cost of this new building, .including ful"'
nishing and equipment, will amount to
.??90.000, towards which the sum ?*
?23 304 in grants and donations has been
received.
A valuable part of the educational work
to be carried on in this new extension Js
the training-school. It will provide fcl
pupils desiring to take the course for the
certificates of the Chartered Society ?'
Massage and Medical Gymnastics.
ments, we know that the authorities
of Guy's have every reason for con-
gratulation on their consistent policy
in respect of the upkeep of the build-
ings, plant, and equipment. This is
a feature of the work, which will
always weigh with the public in the
matter of support. Of the quality of
work, which is all-important, there is
no question, and the record of Guy's
is such as to justify the hope of the
Governors that it may attract the
attention of some generous donor,
who may be moved in his generation
to rival the noble endowments that
Thomas Guy and William Hunt made
in the cause of the charity in the years
1724 and 1829. This hope all who
know Guy's will earnestly echo.
THE NEW MASSAGE BUILDING.
The new building providing out-patient
departments for massage, remedial exer-
cises and electricity, with additional ac-
commodation for nurses, of which we give
an illustration and plans, as designed by
the architect, Mr. Wm. J. Walford,
F.R.I.B.A., is a self-contained unit. It
stands on a portion of the site in the
" Park " formerly covered by the old
clinical wards, Miriam and John, which
were built in 1797. The ground floor pro-
A'ides accommodation for the treatment of
patients by massage and electrical exer-
Scale 16 ft. to tlio inch.
SUBWAY* |1 T GROUND FLOOR.
Scale 16 ft. to the inch.
LOWER GROUND FLOOR.
cises, with the treatment rooms, specially
equipped for the purpose, arranged around
a central waiting-hall. There is a large
gymnasium with a rubber floor for the
instruction of pupils in remedial exercises
and for the treatment of patients, and on
the lower ground floor are whirlpool baths
and a swimming exercises bath. Here
there are also class rooms, a library, and
a pupils' common room. The first floor is
devoted to the treatment of patients by
electricity. The three upper floors pro-
vide additional accommodation for the
nurses. There is an electric passenger lift
to the full height of the building and
all rooms are supplied from the central
power-station of the hospital with heat-
ing, hot water, and electricity for power
and light. The massage wards are laid
with Decolite flooring and the dados are
of the same material. The rubber floor f?r
the gymnasium has been presented by the
Rubber Growers' Association. The total
cost of this new building, .including fur"
nishing and equipment, will amount to
.??90.000. towards which the sum ?*
?23.304 in grants and donations has been
received.
A valuable part of the educational work
to be carried on in this new extension lS
the training-school. It will provide fcl
pupils desiring to take the course for the
certificates of the Chartered Society
Massage and Medical Gymnastics.

				

## Figures and Tables

**Figure f1:**
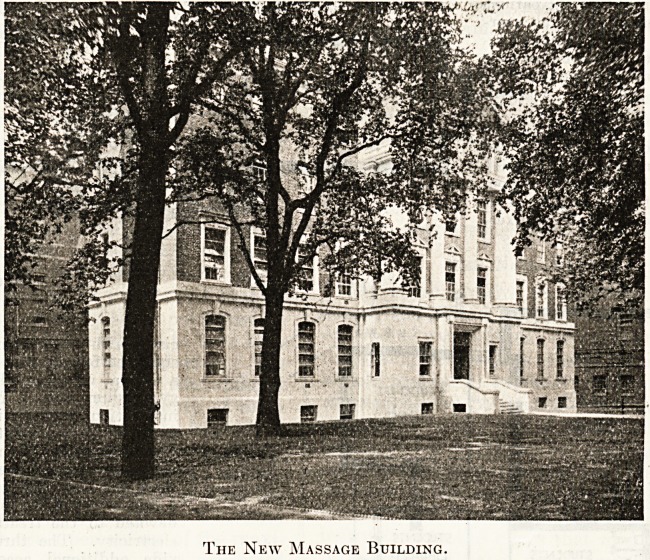


**Figure f2:**